# Familial liability to asthma and ADHD: A Swedish national register‐based study

**DOI:** 10.1002/jcv2.12044

**Published:** 2021-10-23

**Authors:** Shihua Sun, Ralf Kuja‐Halkola, Zheng Chang, Samuele Cortese, Catarina Almqvist, Henrik Larsson

**Affiliations:** ^1^ Department of Medical Epidemiology and Biostatistics Karolinska Institutet Stockholm Sweden; ^2^ Center for Innovation in Mental Health Academic Unit of Psychology University of Southampton Southampton UK; ^3^ Pediatric Allergy and Pulmonology Unit at Astrid Lindgren Children's Hospital Karolinska University Hospital Solna Sweden; ^4^ School of Medical Sciences Örebro University Örebro Sweden

**Keywords:** ADHD, asthma, family co‐aggregation, twin modeling

## Abstract

**Background:**

Studies have reported significant associations between asthma and attention‐deficit/hyperactivity disorder (ADHD), but whether the association is due to shared etiology such as shared genetic risk factors remains unclear. We aimed to investigate patterns of familial co‐aggregation of asthma and ADHD and also to quantify the relative contribution of genetic and environmental influences.

**Methods:**

Through Swedish register linkages, we obtained a cohort of 927,956 individuals born 1992–2001 and identified monozygotic twins (MZ), dizygotic twins (DZ), full‐ and half‐siblings, and full‐ and half‐cousins. Clinical diagnosis of asthma and ADHD were identified from the Swedish national registers. We used logistic regressions to investigate the within‐individual association and familial co‐aggregation between asthma and ADHD. We then used bivariate twin modeling to quantify the genetic and environmental correlations and their contributions to the familial liability.

**Results:**

Individuals with asthma had significantly higher risk of ADHD (odds ratio [OR], 1.50; 95% confidence interval [CI], 1.47–1.54). Relatives of individuals with asthma had an increased risk of ADHD compared to relatives of individuals without asthma; in familial co‐aggregation analysis, the association was strongest in MZ twins (OR, 1.67; 95% CI, 0.99–2.84) and attenuated with degree of genetic relatedness. In the twin modeling, the phenotypic and genetic correlations between asthma and ADHD estimated from the ACE model were 0.09 (95% CI, 0.05–0.14) and 0.12 (95% CI, 0.02–0.21), respectively. The bivariate heritability was 0.88 (95% CI, 0.30–1.46). Estimates for contributions from shared and non‐shared environment factors were not statistically significant.

**Conclusions:**

Asthma and ADHD co‐aggregate in families primarily due to shared genetic risk factors. Within‐individual and family history of either disorder should prompt clinical assessment of the other condition. Future studies should further investigate genetic variants underlying the co‐occurrence of ADHD and asthma.

## Graphical abstract

Asthma and attention deficit/hyperactivity disorder (ADHD) co‐aggregate in families. The comorbidity between asthma and ADHD may be mostly explained by shared genetic risk factors.

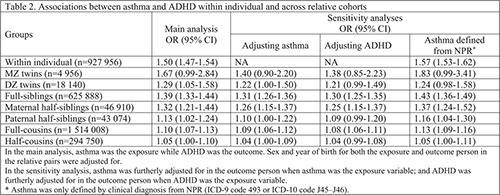

**Key points**

- Previous studies reported a robust association between asthma and attention‐deficit/hyperactivity disorder (ADHD), but the underlying mechanisms remained largely unknown.
- Our analysis revealed that asthma and ADHD co‐aggregate in the family, and the phenotypic correlation between the two disorders may mostly be explained by shared genetics.
- The familial co‐aggregation of asthma and ADHD supports joint services for clinicians with expertise on developmental psychopathology and allergic/atopic conditions to aim for early identification and proper management of either disorders.
- The bivariate heritability and genetic correlation may facilitate future research to identify biological pathways from genetic variants to the specific phenotypes.

## INTRODUCTION

Asthma is the most common chronic respiratory disease with onset in childhood (Stanescu et al., 2019). Studies have reported significant associations between asthma and psychiatric conditions including attention‐deficit/hyperactivity disorder (ADHD) (Wright et al., 1998), which is the most prevalent neurodevelopmental disorder (Posner et al., 2020). A recent meta‐analysis found a significant association between ADHD and asthma, and the association was replicated in national register data even after controlling for a number of possible confounders (Cortese et al., 2018). Although the study provided evidence for a robust link between asthma and ADHD at the population level, the underlying mechanisms (whether one disorder causes another or the two share common causes) are not established.

Asthma and ADHD are both highly heritable disorders, with twin studies estimating the heritability estimates of asthma (Ullemar et al., 2016) and ADHD (Larsson et al., 2014) to be both around 80%. Recently, a large genome‐wide association meta‐analysis (Demontis et al., 2019) of ADHD reported a small genetic correlation with asthma, but the extent to which these findings replicate in family and twin data remains unclear.

Early work from clinically‐based sibling studies (Biederman et al., 1994; Hammerness et al., 2005) found that asthma and ADHD are independently transmitted in families, without shared familial causes. The sample sizes were limited, and boys and girls were analyzed separately. Family co‐aggregation studies with large sample size, incorporating different degrees of relatives are still missing. Large‐scale family co‐aggregation studies incorporating multiple relatives could provide insight into the potential value of using within‐individual or family history information to facilitate earlier identification of asthma and ADHD.

In addition to family studies, twin modeling analysis has been used to quantify the relative contribution of genetic and environmental factors. Two studies (Holmberg et al., 2015; Mogensen et al., 2011) based on the Swedish twin registry, using parent‐rated symptoms of ADHD, found that asthmatic children had increased risk of presenting with ADHD symptoms, but findings regarding the relative contribution of genetic and environmental factors were inconclusive due to limited power. Furthermore, none of the previous studies have conducted twin modeling analysis on clinically diagnosed asthma and ADHD, which constrains possibilities to generalize to clinical populations and to the severe end of the asthma and ADHD symptom distribution.

To fill these gaps, in this study, we used the nationwide Swedish register data aiming (1) to investigate the shared familial liability to asthma and ADHD by analyzing multiple types of relatives, and (2) to conduct the first twin study using clinically diagnosed asthma and ADHD to estimate the relative contribution of genetic and environmental influences to the association.

## MATERIALS AND METHODS

The study was approved by the Regional Ethics Committee in Stockholm Sweden. Since this is a register‐based study using anonymized data, informed consent for study individuals was not required, except for information on zygosity of the twins where consent was obtained from the participants.

### Study population

**FIGURE 1:**
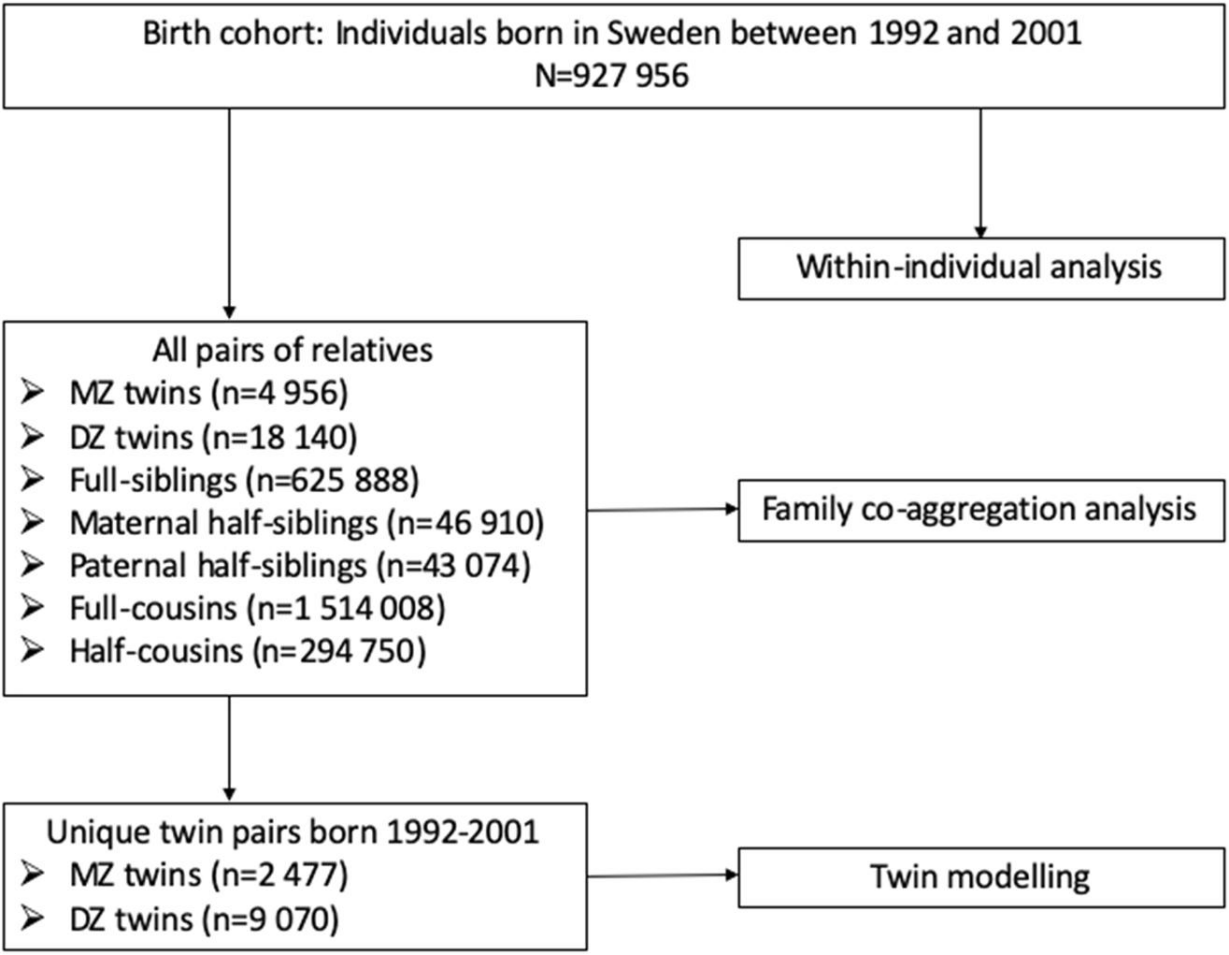
Flow diagram of data extraction process to generate cohorts for the analysis. There was a family with two monozygotic twins twin pairs with only one pair selected for twin modeling, resulting in the unique twin pairs (*n* = 2477) less than half of all twin pairs (*n* = 4956)

Data was identified by linkage through multiple Swedish national registers using unique personal identification numbers. The main cohort included individuals born in Sweden between January 01, 1992 and December 31, 2001, identified from the Medical Birth Register (Axelsson, 2003; Cnattingius et al., 1990). Information on emigration from Sweden and date of death before December 31, 2013 was extracted from the Migration Register and the Cause of Death Register (Brooke et al., 2017), respectively. After excluding deaths and emigration before 3 years old, we obtained a total of 927,956 individuals. Through the Multi Generation Register (Ekbom, 2011) and Swedish Twin Registry (Zagai et al., 2019), each individual was linked to their biological parents and then to establish 7 cohorts of relatives: monozygotic (MZ) twin pairs, dizygotic (DZ) twin pairs, full‐siblings, maternal and paternal half‐siblings, as well as full and half cousins. All possible pairs of siblings and cousins were identified, with each individual contributing at least once as the exposure relative and once as the outcome relative in the main analysis. Information on relative cohorts used in each step of analysis was illustrated in Figure 1.

### Asthma and ADHD identification

Asthma and ADHD were defined after 3 years of age, using the same standards as in our previous study analyzing cross‐sectional associations between the two disorders (Cortese et al., 2018). Asthma was defined as clinical diagnosis from the National Patient Register (NPR) (Ludvigsson et al., 2011) after age three (ICD‐9 code 493 or ICD‐10 code J45–J46) or prescriptions of asthmatic medications (Anatomic Therapeutic Codes R03AC, A03AK, R03BA, and R03DC) from the Prescribed Drug Register (Cortese et al., 2018; Ortqvist et al., 2013). ADHD was defined as the first clinical diagnosis (ICD‐9 code 314 or ICD‐10 code F90) from NPR.

### Statistical analysis

We used logistic regression models to estimate odds ratios (OR) with two‐sided 95% confidence intervals (CIs) for the association between asthma and ADHD on individual level and within the family. For all models, we accounted for dependence of rows of data from within family clusters using cluster‐robust variance estimation to adjust the standard errors.

### Within‐individual analysis

We started by comparing the risks of ADHD among individuals with and without asthma, adjusting for sex and year of birth, to validate the phenotypic association (within‐individual analysis).

### Familial co‐aggregation analysis

We estimated the ORs of having ADHD in individuals (outcome person) whose relatives (exposure person) had asthma compared to individuals whose relatives did not have asthma. We adjusted for sex and year of birth for both the exposure person and outcome person. An increased risk of having ADHD among individuals whose twins, siblings or cousins had asthma indicates the existence of shared familial liability underlying the phenotypic association between asthma and ADHD. Existence of familial liability can be inferred by the different magnitude of associations in different degrees of siblings (Brikell et al., 2018; Ghirardi et al., 2018). Furthermore, higher ORs in MZ twins than other siblings or cousins indicate the existence of genetic contribution to the phenotypic overlap, and higher ORs in maternal half‐siblings compared to paternal half‐siblings indicate shared environmental contributions (Brikell et al., 2018; Yao et al., 2016).

#### Sensitivity analysis

It is possible that the observed familial association may be better explained by causality between the two disorders in the same individual, that is that one disorder directly influences the risk of the other, instead of by shared familial liability. To test this alternative hypothesis, we first repeated the analysis with further adjustment of asthma in the outcome person whose ADHD status was assessed. We then swapped ADHD as the exposure and repeated the analysis adjusting for ADHD in the outcome person whose asthma status was assessed. Estimates remaining consistently similar with the main analysis would furtherly support the existence shared familial liability contributing to the phenotypic association (see a similar study for more detailed explanation of the methodology; Yao et al., 2016), while attenuation of the association may suggest existence of direct causality between asthma and ADHD.

To test the robustness of the asthma definition, we did a set of sensitivity analysis by defining asthma only based on clinical diagnosis from NPR without using information from PDR (same as in the main analysis, asthma was the exposure and ADHD was the outcome).

### Twin modeling analysis

Unique twin pairs were used in this part. We calculated the intra‐class (ICCs, correlations of the same trait across twins) and cross‐twin‐cross‐trait (CTCT, correlations of trait one in sibling one and trait two in sibling two) correlations. We used bivariate twin modeling to estimate the relative genetic, shared‐, and non‐shared environmental contributions to the familial liability and to estimate the genetic correlation between asthma and ADHD. Asthma and ADHD were treated as binary conditions representing underlying normal distribution of liability of getting the diagnosis based on a liability threshold framework (Neale & Cardon, 2013). The potential shared familial liability may consist of contributions from additive genetic factors (A), dominant genetic factors (D), shared environmental (C, non‐genetic factors that make twins in a pair similar), or non‐shared environmental (E, factors unique to the individuals) factors. The broad sense heritability (H) consists of both A and D (H = A + D). We first fitted a saturated model on means, variances and correlations, including sex and year of birth as covariates. Sub‐models including ACE (assuming no dominant genetic factors), ADE (assuming no shared environmental factors) and AE (assuming only additive genetic and non‐shared environmental factors) models were then fitted separately for decomposition of the familial liability. We used likelihood ratio tests to compare performance of the sub‐models with the saturated models. A sub‐model (with less assumptions) with better fitting performance (defined by akaike information criterion, AIC) will be preferred to the saturated model and other sub‐models (Neale & Cardon, 2013; Tan et al., 2015). The following assumptions were considered. Correlation for A is one in MZ twins as they are genetically identical, and 0.5 in DZ twins as they share 50% of their co‐segregation alleles. Correlation for D is 0.5 in MZ twins and 0.25 in DZ twins. Twins correlate 1 for C factors.

Data extraction was done using SAS, version 9.4 (SAS Institute, Inc.), the familial co‐aggregation analysis was done using version 15.1 (StataCorp), and the quantitative genetic modeling part was conducted using OpenMx (Neale et al., 2016) in R.

## RESULTS

As shown in Table 1, we identified in total 927,956 individuals in the main cohort, with 474,462 males (51.13%) and 453,494 females (48.87%). Overall, 121,891 (13.14%; mean age of diagnosis 9.8 ± 4.3) individuals received a diagnosis of asthma and 39,298 (4.23%; mean age of diagnosis 13.3 ± 3.6) were diagnosed with ADHD, with 7334 (0.79%) having both asthma and ADHD. Prevalence of asthma and ADHD was higher in males than in females, and higher proportion of males (4932, 1.04%) were diagnosed with both disorders than females (2402, 0.53%).

**TABLE 1:** Descriptive statistics of the total cohort (1992–2001)

	Total	Asthma	ADHD	Asthma and ADHD
	*N* (column percent)	*N* (row percent)	*N* (row percent)	*N* (row percent)
Overall	927,956 (100.0)	121,891 (13.14)	39,298 (4.23)	7334 (0.79)
Sex				
Male	474,462 (51.13)	67,639 (14.26)	26,489 (5.58)	4932 (1.04)
Female	453,494 (48.87)	54,252 (11.96)	12,809 (2.82)	2402 (0.53)
Year of birth				
1992–1996	513,691 (55.36)	64,752 (12.61)	22,015 (4.29)	3988 (0.78)
1997–2001	414,265 (44.64)	57,139 (13.79)	17,283 (4.17)	3346 (0.81)

Abbreviation: ADHD, attention‐deficit/hyperactivity disorder.

### Within‐individual analysis

Individuals with asthma were at increased risk of also having ADHD (OR, 1.50; 95% CI, 1.47–1.54; Table 2) compared to individuals without asthma.

**TABLE 2:** Associations between asthma and ADHD within individual and across relative cohorts

Groups	Main analysis OR (95% CI)	Sensitivity analyses OR (95% CI)		
		Adjusting asthma	Adjusting ADHD	Asthma defined from NPR^a^
Within individual (*n* = 927,956)	1.50 (1.47–1.54)	NA	NA	1.57 (1.53–1.62)
MZ twins (*n* = 4956)	1.67 (0.99–2.84)	1.40 (0.90–2.20)	1.38 (0.85–2.23)	1.83 (0.99–3.41)
DZ twins (*n* = 18,140)	1.29 (1.05–1.58)	1.22 (1.00–1.50)	1.21 (0.99–1.49)	1.24 (0.98–1.58)
Full‐siblings (*n* = 625,888)	1.39 (1.33–1.44)	1.31 (1.26–1.36)	1.30 (1.25–1.35)	1.43 (1.36–1.49)
Maternal half‐siblings (*n* = 46,910)	1.32 (1.21–1.44)	1.26 (1.15–1.37)	1.25 (1.15–1.37)	1.37 (1.24–1.52)
Paternal half‐siblings (*n* = 43, 074)	1.13 (1.02–1.24)	1.10 (1.00–1.22)	1.09 (0.99–1.20)	1.16 (1.04–1.30)
Full‐cousins (*n* = 1,514,008)	1.10 (1.07–1.13)	1.09 (1.06–1.12)	1.08 (1.06–1.11)	1.13 (1.09–1.16)
Half‐cousins (*n* = 294,750)	1.05 (1.00–1.10)	1.04 (1.00–1.09)	1.04 (0.99–1.08)	1.05 (1.00–1.11)

*Note*: In the main analysis, asthma was the exposure while ADHD was the outcome. Sex and year of birth for both the exposure and outcome person in the relative pairs were adjusted for. In the sensitivity analysis, asthma was furtherly adjusted for in the outcome person when asthma was the exposure variable; and ADHD was furtherly adjusted for in the outcome person when ADHD was the exposure variable.

Abbreviations: ADHD, attention‐deficit/hyperactivity disorder; CI, confidence interval; DZ, dizygotic twins; MZ, monozygotic twins; NPR, National Patient Register; OR, odd ratio.

^a^ Asthma was only defined by clinical diagnosis from NPR (ICD‐9 code 493 or ICD‐10 code J45–J46).

### Family co‐aggregation analysis

Individuals whose relatives had asthma presented with higher risk of having ADHD compared to individuals whose relatives did not have asthma (Table 2). The association among MZ twins (OR, 1.67; 95% CI, 0.99–2.84) was stronger than the within‐individual association, and was strongest across the relative cohorts, although not statistically significant due to potentially limited power. The associations were all statistically significant for DZ pairs, full‐ and half‐sibling pairs, and full‐ and half‐cousins where we had more power. Associations between asthma and ADHD gradually attenuated as a function of the degree of the genetic relatedness, indicating contributions from shared genetics to the observed associations. Odds ratios estimated from maternal half‐siblings were slightly higher than paternal half‐siblings, indicating possibility of contributions from shared environmental factors, although the confidence intervals largely overlapped.

In the sensitivity analysis while adjusting for the exposure disorder status in the outcome person (Table 2), the associations partly attenuated from the main analysis but the pattern across relative cohorts remained the same. In the analysis treating asthma as the exposure disorder, results were similar to the ones in the analysis treating ADHD as the exposure, further supporting the existence of underlying familial liability. Moreover, ORs estimated when asthma was only defined by clinical diagnosis from NPR were similar with estimates from the main analysis.

### Twin modeling analysis

Table 3 shows the number of concordant and discordant pairs for asthma and ADHD, as well as the observed tetrachoric correlations in twins born between 1992 and 2001. ICCs for asthma and ADHD were higher among MZ twins than DZ twins. CTCT was also higher among MZ twins (0.13; 95% CI, 0.07–0.19) than DZ twins (0.08; 0.03–0.13).

**TABLE 3:** Concordant versus discordant pairs and tetrachoric correlations across siblings

	Within trait				Cross traits			
	Asthma		ADHD			Tetrachoric correlations (95% CI)		
	Both affected/both unaffected^b^	Discordant pairs^c^	Both affected/both unaffected	Discordant pairs	Concordant pairs^d^	ICC asthma	ICC ADHD	CTCT
All pairs^a^	506/8955	2086	129/10,795	623	75	–	–	–
MZ twins	146/2057	274	42/2377	58	14	0.74 (0.69–0.80)	0.90 (0.85–0.96)	0.13 (0.07–0.19)
DZ twins	360/6898	1812	87/8418	565	61	0.33 (0.29–0.38)	0.53 (0.47–0.60)	0.08 (0.03–0.13)

Abbreviations: ADHD, attention‐deficit/hyperactivity disorder; CI, confidence interval; CTCT, cross‐twin‐cross‐trait; DZ, dizygotic twins; MZ, monozygotic twins; OR, odd ratio.

^a^ Unique sibling pairs for quantitative genetic modeling.

^b^ Number of pairs with both siblings affected versus (/) number of pairs with both siblings unaffected by the disorder.

^c^ Number of pairs where one sibling was affected with the disorder and the other was unaffected.

^d^ Number of pairs where one sister was affected with asthma and the other affected with ADHD.

Estimates from the bivariate ACE, ADE and AE models are presented in Table 4. Overall, ACE model was the best fitting model with the lowest AIC, and with non‐deterioration of model fit as indicated from likelihood ratio test. The univariate heritability for asthma was similar across the three models, but heritability for ADHD from ADE model (0.90; 0.86–0.95) and AE model (0.90; 0.86–0.95) was higher than the estimate from ACE model (0.70; 0.66–0.77). Non‐shared environmental factors were estimated to be significantly positive for within‐trait variances for both asthma and ADHD.

**TABLE 4:** Estimates from bivariate quantitative genetic analysis

	ACE model	ADE model	AE model
Coefficients^a^	Estimates (95% CI)	Estimates (95% CI)	Estimates (95% CI)
Degree of freedom	15	15	12
AIC	−67,943.98	−67,938.17	−67,943.20
rPh	0.09 (0.05–0.14)	0.09 (0.05–0.13)	0.09 (0.05–0.14)
A for asthma	0.71 (0.66–0.77)	0.63 (0.56–0.70)	0.72 (0.68–0.76)
A for ADHD	0.70 (0.53–0.86)	0.90 (0.86–0.94)	0.90 (0.86–0.95)
D for asthma	0.00 (0.00–0.00)	0.10 (0.06–0.15)	0.00 (0.00–0.00)
D for ADHD	0.00 (0.00–0.00)	0.00 (NA)	0.00 (0.00–0.00)
H for asthma	0.71 (0.66–0.77)	0.73 (0.69–0.78)	0.72 (0.68–0.76)
H for ADHD	0.70 (0.53–0.86)	0.90 (0.86–0.95)	0.90 (0.86–0.95)
C for asthma	0.00 (−0.01–0.02)	0.00 (0.00–0.00)	0.00 (0.00–0.00)
C for ADHD	0.19 (0.05–0.32)	0.00 (0.00–0.00)	0.00 (0.00–0.00)
E for asthma	0.28 (0.23–0.33)	0.27 (0.22–0.31)	0.28 (0.24–0.32)
E for ADHD	0.12 (0.06–0.17)	0.10 (0.05–0.14)	0.10 (0.05–0.14)
rA	0.12 (0.02–0.21)	0.16 (0.13–0.19)	0.14 (0.06–0.22)
rD	NA^b^	−1.00 (−1.00–1.00)	NA
rH	0.12 (0.02–0.21)	0.13 (0.01–0.26)	0.14 (0.06–0.22)
rC	1.00 (1.00–1.00)	NA	NA
rE	−0.09 (−0.32–0.15)	−0.11 (−0.72–0.51)	−0.13 (−0.46–0.21)
Bivariate A	0.88 (0.30–1.46)	1.34 (0.33–2.35)	1.22 (0.59–1.85)
Bivariate D	0.00 (0.00–0.00)	−0.16 (NA)	0.00 (0.00–0.00)
Bivariate H	0.88 (0.30–1.46)	1.18 (0.11–2.25)	1.22 (0.59–1.85)
Bivariate C	0.29 (−0.34–0.91)	0.00 (0.00–0.00)	0.00 (0.00–0.00)
Bivariate E	−0.17 (−0.66–0.33)	−0.18 (−1.25–0.89)	−0.22 (−0.85–0.41)

Abbreviations: ADHD, attention‐deficit/hyperactivity disorder; AIC, Akaike information criterion; CI, confidence interval; DZ, dizygotic twins; MZ, monozygotic twins; rPh: phenotypic correlation.

^a^ rA/D/H/C/E: additive/dominant/overall genetic correlations and shared/non‐shared environmental correlations. Bivariate A/D/H/C/E: proportions of the phenotypic correlation explained by A/D/H/C/E.

^b^ NA: Unavailable or without interpretable confidence intervals.

Estimated phenotypic correlations and genetic correlations were similar across the three models; with the ACE model presenting a phenotypic correlation of 0.09 (0.05–0.14) and a genetic correlation of 0.12 (0.02–0.21). Shared genetic factors explained almost all of the covariance between asthma and ADHD, with bivariate heritability of 0.88 (0.30–1.46) estimated from the ACE model. Correlation coefficients for E was not statistically significant (−0.09; −0.32–0.15). None of the models supported significant contributions from shared or non‐shared environmental factors for the phenotypic correlation.

## DISCUSSION

To our knowledge, this is the largest study using family data from about 1 million individuals to investigate the familial liability underlying the association between clinically diagnosed asthma and ADHD. It is also the first to quantify the relative contribution of genetic and environmental influences to the familial liability using twin modeling analysis. We found that individuals with asthma were at a higher risk of having ADHD compared to those without asthma. The association was strongest in MZ twins, then attenuated with degree of genetic closeness and the pattern was mostly explained by a shared familial liability. Using twin analyses, we also found that a substantial part of the association between asthma and ADHD were explained by genetic factors.

We found, for the first time, that relatives of probands diagnosed with either asthma or ADHD have elevated risk of being diagnosed with the other. Two early sibling studies did not find evidence of familial co‐aggregation between asthma and ADHD (Biederman et al., 1994; Hammerness et al., 2005), probably due to limited power. Our finding of a link between asthma and ADHD within individuals and across relatives highlights the need for clinicians who see young patients with asthma to carefully query about problems of inattention, hyperactivity, and impulsivity that may suggest the need of a referral to a child psychiatrist/psychologist or behavioral pediatrician with expertise in ADHD. It also points to the importance of joint services where clinicians with expertise in developmental psychopathology and those specialized in allergic/atopic conditions may quickly and effectively interact to provide timely and high‐quality care to patients with the double burden of developmental disorders (such as ADHD) and medical conditions (such as asthma). Of note, practitioners dealing with asthmatic children and not aware of the link between asthma and ADHD may consider comorbid inattention, hyperactivity and impulsivity as asthma symptomatology or results from asthma treatment (Hammerness et al., 2005), or a consequence of a psychological distress due to the fact of having a chronic medical condition (Borschuk et al., 2018; Daly et al., 1996; Schmitt et al., 2010), thus missing an important opportunity to identify a neuropsychiatric condition for which effective treatments are available (Chan et al., 2016; Chang et al., 2019; Cortese, 2020). On the other end, our findings also suggest that practitioners seeing patients referred for ADHD, should query about personal and family history of symptoms of asthma, which may pin to the need of a prompt referral for specialist assessment of asthma, thus reducing the diagnostic delay for this condition.

Results from our twin modeling analysis indicated that the association between asthma and ADHD may be substantially explained by shared etiology, especially shared genetic risk factors, which was also supported by the pattern of associations across relatives in our familial‐coaggregation analysis. The overall phenotypic and genetic correlations between asthma and ADHD were weak but statistically significant. A recent study investigating the associations of polygenic risk scores for either condition and the other disorder reported non‐significant results, but the findings were based on a small sample size and self‐reported asthma diagnosis (Leffa et al., 2021). The largest GWAS on ADHD (Demontis et al., 2019) to date investigated genetic correlations between ADHD and 219 phenotypes using summary statistics, and reported a genetic correlation between asthma and ADHD of 0.17, which was comparable to estimates in our twin modeling analysis (in ACE model it was 0.12; 0.02–0.21), and similar with another summary statistics analysis (genome‐wide genetic correlation 0.197) based on UK Biobank and the Psychiatric Genomics Consortium (PGC) (Zhu et al., 2019). Similar estimates from twin studies and studies using contemporary genome‐wide approaches may indicate that a large part of the genetic correlation between asthma and ADHD is associated with common variants captured by SNP genotyping. Two studies based on parent‐reported asthma and ADHD from the Swedish Twin Registry, one of which is partly overlapping with the current study, reported a robust phenotypic correlation between asthma and symptoms including hyperactivity/impulsivity and inattention (Holmberg et al., 2015; Mogensen et al., 2011). Similar with our findings, results from both previous twin studies indicated that the phenotypic correlation might be mostly explained by shared genetics, although the genetic correlation seemed to be weak. The estimated heritability of asthma and ADHD in our data were comparable with previous reports (Bijanzadeh et al., 2011; Faraone & Larsson, 2019; Ullemar et al., 2016), consistently supporting that asthma and ADHD are highly heritable. The size of the genetic correlation between these two highly heritable conditions indicate that only a small proportion of the total genetic underpinnings of asthma and ADHD is shared across disorders. Nonetheless, our findings provided significant evidence that the comorbidity between the most commonly diagnosed respiratory disease and the most common neurodevelopmental disorder may be largely due to common causes. Future studies using designs such as polygenic risk scores or biological pathway analysis with appropriate samples are needed to further clarify if asthma and ADHD share genetic pathways or if the genetic sharing reflects mediation (i.e., vertical pleiotropy). For example, studies hypothesized that asthma may predispose higher risk of subsequent ADHD (Blackman & Gurka, 2007; Chen et al., 2013; Mogensen et al., 2011), possibly mediated by hyper‐secretion of pro‐inflammatory cytokines from allergic reactions passing through the blood‐brain barrier and affecting the prefrontal cortex (Buske‐Kirschbaum et al., 2013; Chen et al., 2013). Families of genes related to the pathogenesis of inflammatory conditions including asthma (Kim & Ober, 2019), and genes associated with ADHD by regulating neurotransmitters such as dopamine (Demontis et al., 2019), could be promising candidates to explore their functions involved with the phenotypic correlation between asthma and ADHD.

It remains to be established whether and in what form there is direct causality between clinically diagnosed asthma and ADHD. The recent study based on summary statistics data from UK Biobank and PGC reported a positive causal effect from ADHD on asthma using Mendelian randomization analysis, but no significant causality on the other direction (Zhu et al., 2019). However, the cohorts included in PGC generating genetic data of ADHD were predominantly children while the cohorts in UK Biobank generating data of asthma were mostly adults. Furthermore, findings from Mendelian randomization analysis, as the authors clarified in the paper, should always be interpreted with caution due to the difficulty of scrutinizing the required assumptions (Davey Smith & Ebrahim, 2003; Smith & Ebrahim, 2004). On the other hand, previous population‐based studies on asthma and ADHD could not provide concrete evidence on the causality of the association either due to cross‐sectional design or failure in addressing important confounding (Cortese et al., 2018). In the current study, we observed attenuations of the ORs when adjusting for the exposure phenotype, indicating the possibility of bi‐directional associations between asthma and ADHD. However, the average diagnosis age of asthma (9.8 ± 4.3) was much younger than ADHD (13.3 ± 3.6). Instead of asthma causing later ADHD, this may rather reflect a delay of diagnosis for ADHD in the Swedish register data, as ADHD may onset before age 12 according to DSM‐5 (Faraone et al., 2015; Posner et al., 2020). Future longitudinal studies with more accurate identification of the timing of both disorders, or Mendelian randomization studies with more appropriate samples and established genetic instrumental variants, may be needed to further clarify the direct causality between incidence of asthma and ADHD.

Findings from our study should be interpreted in light of both its strengths and limitations. First, our data was extracted from national linkages with large sample size in different relative cohorts, resulting in robustness and generalizability of our results. Second, asthma and ADHD were identified from clinical records, and our results were therefore with clinical implications. Although the criteria of combining clinical diagnosis and prescription of asthmatic medications were validated, we could not completely rule out the possibility of prescriptions due to indications other than asthma (such as wheezing due to viral infection among children). However, results from the sensitivity analysis when defining asthma only by clinical diagnosis from NPR were similar with the main analysis, indicating robustness of our asthma definition. The overall phenotypic correlation between asthma and ADHD was weak, which may make it hard for us to conclude on the environmental contributions. We did observe attenuated ORs when adjusting for asthma or ADHD in the outcome person in the familial co‐aggregation analysis and a weak and non‐significant correlation for non‐shared environmental factors in the ACE model, indicating possibly weak causality between asthma and ADHD. Future twin modeling studies with larger sample size may be able to further clarify this issue.

## CONCLUSIONS

Asthma and ADHD co‐aggregate in families, and the familial liability may mostly be explained by genetic factors. Our results have important implication for the clinical practice and the understating of the pathophysiology for both disorders. Asthmatic patients with personal or family history of ADHD symptoms should be considered to be referred for assessment of ADHD, and children with ADHD assessed for asthma. Future studies should further investigate genetic variants explaining the genetic correlation between asthma and ADHD, and clarify the biological pathways from genes to the phenotypic correlation.

## CONFLICT OF INTERESTS

Henrik Larsson has served as speaker for Evalon and Shire and has received research grants from Shire. He is also Editor‐in‐Chief of JCPP *Advances*. The remaining authors report no financial relationships with commercial interests. Samuele Cortese declares reimbursement for travel and accommodation expenses from the Association for Child and Adolescent Central Health (ACAMH) in relation to lectures delivered for ACAMH, Canadian ADHD Alliance Resource (CADDRA), British Association of Psychopharmacology (BAP), and from Healthcare Convention for educational activity on ADHD. He is a member of the Editorial Advisory Board for JCPP *Advances*. Zheng Chang is also a member of the Editorial Advisory Board for JCPP *Advances*. The remaining authors have declared that they have no competing or potential conflicts of interest. [Corrections made on 22 June 2022, after first online publication: This Conflict of Interests statement has been updated in this version.]

## ETHICS STATEMENT

The study was approved by the Regional Ethics Committee in Stockholm Sweden. Since this is a register‐based study using anonymized data, informed consent for study individuals was not required, except for information on zygosity of the twins where consent was obtained from the participants.

## AUTHOR CONTRIBUTIONS

S. Sun and H. Larsson were responsible for the study design. S. Sun and R. Kuja‐Halkola conducted the analysis. S. Sun drafted the manuscript and all co‐authors contributed in the study by providing ideas and comments on the manuscript.

## Data Availability

Dr. Shihua Sun and Dr. Henrik Larsson have full access to the data analysed in the current study.
